# Animal models of diabetic retinopathy: doors to investigate pathogenesis and potential therapeutics

**DOI:** 10.1186/1423-0127-20-38

**Published:** 2013-06-20

**Authors:** Dong Hyun Jo, Chang Sik Cho, Jin Hyoung Kim, Hyoung Oh Jun, Jeong Hun Kim

**Affiliations:** 1 Fight against Angiogenesis-Related Blindness (FARB) Laboratory, Clinical Research Institute, Seoul National University, Seoul 110-744, Republic of Korea; 2 Department of Biomedical Sciences, College of Medicine, Seoul National University, Seoul 110-799, Republic of Korea; 3 Department of Ophthalmology, College of Medicine, Seoul National University, Seoul 110-744, Republic of Korea

**Keywords:** Animal model, Diabetic retinopathy, Macular edema, Pathologic angiogenesis, Vascular permeabilit

## Abstract

Effective and validated animal models are valuable to investigate the pathogenesis and potential therapeutics for human diseases. There is much concern for diabetic retinopathy (DR) in that it affects substantial number of working population all around the world, resulting in visual deterioration and social deprivation. In this review, we discuss animal models of DR based on different species of animals from zebrafish to monkeys and prerequisites for animal models. Despite criticisms on imprudent use of laboratory animals, we hope that animal models of DR will be appropriately utilized to deepen our understanding on the pathogenesis of DR and to support our struggle to find novel therapeutics against catastrophic visual loss from DR.

## Review

### Introduction

Systemic control of blood glucose can slow down the progression of diabetic retinopathy (DR), but fails to stop or reverse clinical signs of DR [1,2]. Furthermore, although general mechanisms of DR are yet to be elucidated, researchers have discovered various molecular pathways regarding DR. In this regard, local treatment options as well as systemic therapeutics of pathogenesis-based approaches are desperately required to prevent catastrophic visual loss from vision-threatening complications of DR such as macular edema (ME), vitreous hemorrhage (VH), and tractional retinal detachment (TRD). Despite criticisms on imprudent uses of laboratory animals, validated animal models are valuable tools to deepen our understanding on the pathogenesis of human diseases and estimate the therapeutic potential of candidate drugs.

As for DR, there are several animal models based on different species of animals from zebrafish to monkeys. Some are based on hyperglycemia that occurs spontaneously or is induced by chemical agents or surgical operation; others mimic clinical consequences of DR and share the molecular pathogenesis of DR. Unfortunately, currently available animal models have limitations in that they only show certain aspects of DR and do not reflect human pathology exactly in the view of retinal structures and mediating factors. Therefore, researchers who utilize animal models of DR should choose animal models that suit for purposes of their research.

In this review, we provide a summary of pathologic features of DR and therapeutic goals in the treatment of DR for further discussion on the selection of appropriate animal models of DR. Then, animal models of DR based on various animals including zebrafish, mice, rats, dogs, and monkeys are listed with details on brief protocols for selected animal models. We hope that this review will help researchers to select appropriate animal models in the investigation of pathogenesis and therapeutic approaches regarding DR.

### Pathologic changes in patients with DR

**Figure 1:**
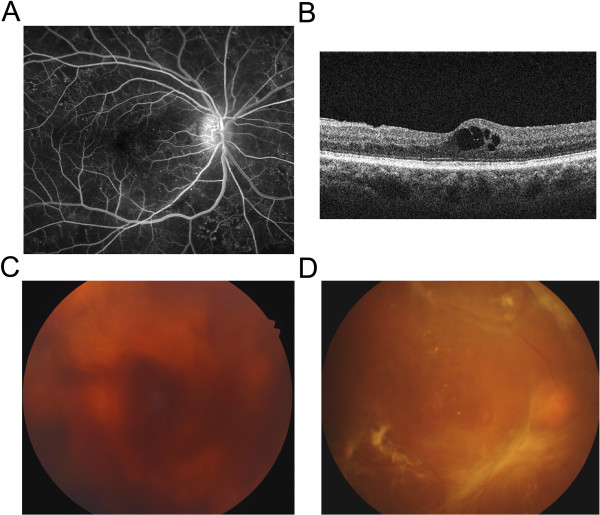
**Pathologic changes in patients with DR.** (**A**) Microaneurysm is one of the characteristic findings in patients with NPDR, demonstrated by tiny hyperfluorescent spots in the fluorescein angiography. (**B**) Disruption in neurovascular units and breakdown of inner BRB leads to ME in DR patients. Optical coherence tomography demonstrates cystoid ME. (**C**) In PDR patients, VH can occur from fragile new vessels. (**D**) Fibrovascular proliferation from retinal neovascularization forms diffuse tractional membrane, resulting in TRD.

Largely, DR is divided into 2 subgroups, nonproliferative DR (NPDR) and proliferative DR (PDR), according to the presence of retinal neovascularization. That is, PDR indicates the condition with retinal neovascularization and accompanying complications from it. In addition, the Early Treatment of Diabetes Retinopathy Study (ETDRS) defined mild, moderate, severe NPDR, and PDR, based on presence of structural abnormalities in the retina, the degree of retinal neovascularization, and accompanying vitreous and/or preretinal hemorrhage [3]. Furthermore, the more practical scale was devised with the sponsorship provided by American Academy of Ophthalmology, which has been utilized widely in everyday clinical settings [4]. In this scale, mild NPDR indicates the condition that there are only microaneurysms in the retina. Severe NPDR is characterized by any of the following retinal abnormalities: 1) more than 20 intraretinal hemorrhages in each of 4 quadrants of the retina, 2) definite venous beading in 2 or more quadrants of the retina, and 3) prominent intraretinal microvascular abnormalities (IRMA) in 1 or more quadrant of the retina. Moderate NPDR is defined as the condition between mild and severe NPDR. Pathologic changes in patients with DR are demonstrated in Figure 1.

#### *Alterations in retinal structures*

In clinical settings, the severity of NPDR is determined by the presence of alterations in retinal structures such as microaneurysms, retinal hemorrhages, venous beading, and IRMA. Microaneurysms are known to occur with thickening of the capillary endothelial basement membrane (BM) and pericyte loss induced by hyperglycemia [5]. This is because many researchers paid attention to corresponding structural abnormalities in various animal models of DR [6]. Venous beading, the manifestation of focal areas of venous dilatation, and IRMA, demonstrated with tortuous intraretinal vessels, indicates the progression of DR clinically and at the same time reflects ischemia and alterations in retinal structures in the view of pathologic conditions. Interestingly, these structural abnormalities progress with time [7]; therefore, they are appropriate barometers for DR as well as clinical signs which require great attention for clinicians who treat patients with DR.

#### *Disruption of neurovascular units: breakdown of blood-retinal barrier (BRB) to ME*

The macula is the central concave area of the retina of which diameter is 5,000 μm. The concavity of the macula helps photoreceptor cells in the central retina to receive the light more effectively than those in surrounding areas. In contrast, patients with ME lose this concavity in the macular area, resulting in blurred vision. In DR, even before the severity reaches the level of PDR, ME can occur to jeopardize the clarity of the vision. Especially, ME located in the most central area of the retina is called clinically significant ME (CSME) [8]. CSME is defined by the ETDRS research group as follows: 1) retinal thickening located at or within 500 μm of the center of the macula, 2) hard exudates at or within 500 μm of the center of the macula with thickening of adjacent retina, or 3) a zone of retinal thickening larger than 1 disc area if located within 1 disc diameter (1,500 μm) of the center of the macula [9].

ME is the phenomenon induced by the disruption of inner BRB, the endothelial barrier constructed by an extensive network of neurovascular units [10]. Neurovascular units indicate robust interactions among neurons, glial cells including astrocytes, periendothelial cells such as pericytes, and endothelial cells to allow the functional integrity of neurons and the formation of blood-neural barriers, BRB and blood–brain barrier [10,11]. Recently, much effort is focused in the development of therapeutic agents which improve the integrity of neurovascular units in central nervous system such as Alzheimer’s disease, stroke, and Parkinson’s disease [11]. Interestingly, most candidate drugs for DR, currently on clinical trials, also mainly target ME [12]. Because various mechanisms regarding DR are involved in the disruption of inner BRB, therapeutics targeting these pathologic pathways might help to reduce visual deterioration from ME. Mechanisms regarding the pathogenesis of DR include hypoxia, inflammation, oxidative stress, and growth factors such as vascular endothelial growth factor (VEGF) [12,13]. These factors influence the vascular permeability and further induce leakage of serum constituents into retinal tissues, resulting in ME.

#### *Pathologic angiogenesis to VH and TRD*

In PDR, ME also occurs from progressive and chronic deterioration in the integrity of the endothelial barrier system. Furthermore, retinal neovascularization from the retinal vasculature in inner retinal layers extends into the vitreous cavity [14]. Like pathologic angiogenesis in the tumor vasculature, retinal neovascularization in DR tends to be fragile and leaky, which can induce further complications from it [15]. In this regard, ruptured neovascularization results in VH, preventing the light from objects to reach the neurosensory retina. In addition, extracellular matrix proteins and fibrous materials can accumulate around new vessels in the retina; this fibrogenic reaction is accelerated by growth factors such as transforming growth factor-β [16]. Unfortunately, fibrovascular tissues formed in the progression of PDR evolve into tractional membrane which can pull the neurosensory retina from underlying tissues, resulting in TRD. Especially, TRD involving the central retina induce visual disturbances in patients with PDR.

### Concerns in care for patients with DR: pathogenesis-based treatment

Advancements in our knowledge of the pathogenesis of DR should lead to the development of pathogenesis-based treatment for the prevention of vision loss in the patients with DR. Unfortunately, current treatment options for DR do not fulfill these tasks effectively in that they only prevent further complications and fail to reverse already occurred structural abnormalities or address only a part of the pathogenesis of DR [12,13]. Possibly, ongoing research with animal models of DR might enable pathogenesis-based treatment options to be utilized in everyday clinical settings.

#### *Drawbacks of current treatment options*

Currently, patients with DR receive combinations of treatment options including systemic control of blood glucose and pressure, surgery, laser photocoagulation, and intravitreal injection of steroid or anti-VEGF agents [11-13]. Although good glycemic control is one of prerequisites in the care of DR patients, clinical signs of DR can progress and sometimes result in vision-threatening complications even after intensive hypoglycemic treatment [1,2]. Therefore, local treatment options are important to prevent progressive visual deterioration from DR. Panretinal photocoagulation (PRP) is performed in patients with high-risk characteristics of PDR and high probability to advance to high-risk PDR [17,18]. High-risk characteristics include following features: 1) new vessels that are larger than one-third disc area and within 1 disc diameter from the optic nerve head (new vessels at the optic disc, NVD) and 2) vitreous or preretinal hemorrhage with NVD less than criteria 1) or new vessels elsewhere that are larger than one-quarter of disc area [3]. Definitely, PRP prevents the occurrence of further complications from DR; however, PRP is a relatively destructive procedure in that it sacrifices the peripheral retina with the purpose of saving the central vision. Likewise, focal photocoagulation, performed for the patients with ME, also blocks further leakage to decrease the frequency of persistent ME, but induces minor visual field losses [9,19]. Furthermore, despite of the preventive potential for the progression of complications, laser photocoagulation has limitations in that it only addresses the consequences of pathologic events. Recently, peeling of inner limiting membrane is performed for the patients with ME to lower mechanical forces regarding the formation of ME [20]. However, without the control of vascular permeability, the effect of surgery on the visual outcome is not substantial. Still, surgery is reserved for the patients with refractory VH or severe TRD involving the fovea [21].

Intravitreal injection of therapeutic agents such as steroids and anti-VEGF agents is relatively invasive compared to laser photocoagulation or systemic hypoglycemic therapy. Intravitreal administration of triamcinolone acetate (IVTA) fails to show a long-term benefit compared to focal photocoagulation in patients with ME [19]. Furthermore, most patients who received IVTA treatment are likely to undergo cataract surgery. In contrast, in patients with pseudophakia, IVTA is still one of therapeutic options for ME because this addresses broadly on inflammatory natures of ME by suppression of inflammatory mediators [12]. Intravitreal injection of anti-VEGF agents such as ranibizumab (Genetech, Inc., South San Francisco, CA, USA), pagaptanib (Eyetech Pharmaceuticals, Inc., Lexington, MA, USA), aflibercept (Regeneron Pharmaceuticals, Inc., Tarrytown, NY, USA), and bevacizumab (Genetech, Inc.) effectively suppresses complications from choroidal neovascularization in wet age-related macular degeneration (AMD) and shows beneficial effects in visual outcomes [13]. Also in DR, these drugs demonstrate positive efficacy on suppression of ME and restoration of vision [22-25]. However, chronic and relapsing nature of ME in DR requires multiple intravitreal injections (over 5 times) per year; therefore, more stable and effective therapeutic agents are still required for the treatment of DR. Furthermore, VEGF inhibition basically has a risk of complications in that VEGF not only induces pathologic angiogenesis and hyperpermeability but also acts as trophic factors for neuronal cells and normal endothelial cells [26-28]. In this regard, pathogenesis-based therapeutic options are to be developed to optimize our armamentarium against DR and minimize potential toxicities; appropriate use of animal models of DR will accelerate these development processes effectively. Currently, bevacizumab is utilized as the off-label drug; the approved indication of pegaptanib and aflibercept does not include DR, but AMD; and the approved indications of ranibizumab include both AMD and DR.

#### *Therapeutic goals in treatment of DR patients*

To overcome drawbacks of current treatment options, researchers might bear in mind the therapeutic goals in the treatment of DR. First of all, the restoration of functional integrity of the retina is important. This objective includes restoration of neurovascular units and the integrity of BRB. As previously mentioned, neurovascular units cover various types of cells and interactions among them via cell to cell contacts and secretory mediators [10,11]. As DR progresses, normal cellular communication is altered with increased level of growth factors such as VEGF and inflammatory cytokines [11]. Further disease progression leads to disruption of tight junction proteins in endothelial cells, resulting in breakdown of inner BRB [29]. In this regard, detailed investigation of specific roles of different kinds of cells and mediators in animal models will definitely help the development of more pathogenesis-based therapeutic options for ME.

Pathologic angiogenesis, that is, retinal neovascularization, is another therapeutic target of DR. Interestingly, retinal new vessels progress with close contact with components of vitreous cavity [14]. Therefore, to treat retinal neovascularization and prevent complications from it such as VH and TRD, both pathologic angiogenesis and interaction between endothelial cells and vitreous proteins can be targeted. In this regard, anti-VEGF agents are utilized to minimize the events of post-vitrectomy VH in PDR patients [30]. Furthermore, the therapeutic approach addressing interaction between endothelial cells and fibronectin in vitreous cavity was investigated to suggest the potential of a different treatment approach [14]. We expect that animal models of retinal neovascularization from inner retinal layers will promote this kind of advancement in the development of novel therapeutics based on different molecules and approaches.

### Prerequisites for effective and validated animal models of DR

In prior sections, we review pathologic changes in patients with DR and therapeutic approaches to treat these patients. Currently, validated animal models are at the center of the development process of novel therapeutic agents and modalities. Before clinical trials, candidate drugs undergo scrutinization through in vitro assays and in vivo animal models. Although there is great need for the development of the intermediate stage of disease-mimicking chips to minimize uncontrolled use of laboratory animals, assays between in vitro assays and in vivo animal models are still lacking. Therefore, we cannot help resorting to validated animal models in the research of pathogenesis and therapeutic applications of DR.

To minimize the imprudent use of laboratory animals and at the same time obtain reproducible results, validated animal models of DR should show following characteristics as prerequisites: 1) Reflecting human pathology. As for structural abnormalities such as microaneurysm and accompanying functional abnormalities such as increased permeability, researchers expect those features to be reproducibly manifested in validated animal models. 2) Easy maintenance of animals. Too large or too weak animals require special care for the maintenance. These animals are not suitable for repeated experiments with stable and reproducible results. 3) Easy induction of diabetic or pathologic conditions. Animal models based on complicated surgery or long-term feeding can promote research on those specific treatment options, but hinder widespread use of animal models. 4) Validated methods of data interpretation. Especially in the models for the evaluation of therapeutic effects, findings should be analyzed statistically to demonstrate therapeutic potential of candidate drugs. In this process, proper randomization of laboratory animals and handling of data are mandatory to optimize the predictive value of studies [31].

### Animal models of DR: from zebrafish to monkeys

As research on diabetes is actively performed, there are several validated animal models for diabetes and some of them are utilized for the investigation of DR. These animal models basically demonstrate induced hyperglycemia as the main characteristic of animal models. In other words, the elevation of serum glucose that is spontaneously, chemically, or surgically induced leads to pathologic events similar to those in patients with diabetes and DR. As previously mentioned, animal models of this category show the pathologic features regarding structural abnormalities or the dissociation of neurovascular units of the retina: 1) pericyte loss, 2) thickening of BM, 3) acellular capillaries or capillary dropout, and 4) increased vascular permeability. Unfortunately, retinal neovascularization in DR is not definite in animal models of DR with induced hyperglycemia [6]. In this regard, researchers have utilized animal models of retinal neovascularization for studying the therapeutic potential of candidate drugs on the status of PDR [14,32,33]. Furthermore, intravitreal and subretinal injection of angiogenic factors such as VEGF also leads to retinal neovascularization or increased vascular permeability [34,35]. In following sections, we review various animal models of DR based on different species of animals from zebrafish to monkeys. Characteristics of each animal model are summarized in Table 1.

**Table 1:** A list of animal models of DR that are based on various species from zebrafish to monkeys

**Animal model**	**Method of induction**	**Type of diabetes**	**Characteristic findings**	**References**
**Zebrafish**				
High glucose treatment	Alternate immersing in glucose/water solution	Type 1	- Thinning of IPL and INL	[38,39]
			- Degeneration of PL	
			- BM thickening in retinal capillaries	
STZ	Intraperitoneal injection of STZ	Type 1	- Thinning of IPL and PL	[46]
Hypoxia	Immersing in hypoxic chamber (10%)	N/A	- New vascular branches and sprouts	[51,52]
**Mice and rats**				
STZ	Intraperitoneal injection of STZ	Type 1	- BM thickening in retinal capillaries	[29,35,41-45,53]
			- Apoptosis of neuronal cells in GCL	
			- Loss of pericytes in retinal capillaries	
			- Loss of amacrine cells in INL	
			- Increased vascular permeability	
db/db mice	Spontaneous hyperglycemia	Type 2	- BM thickening in retinal capillaries	[54-59]
			- Loss of pericytes	
			- Apoptosis of neuroretinal cells	
			- Increased vascular permeability	
Non-obese diabetic mice	Spontaneous hyperglycemia	Type 1	- Apoptosis of ganglion cells in GCL	[61,62]
			- Abnormal focal vascular proliferation	
Akita mice	Spontaneous hyperglycemia	Type 1	- Apoptosis of neuronal cells in GCL	[45,63,64]
			- Loss of amacrine cells in INL	
			- Acellular capillaries	
			- Loss of pericytes	
			- Thinning of IPL and INL	
			- Increased vascular permeability	
			- Microaneurysm formation	
			- New vessels in OPL	
Zucker diabetic fatty rats	Spontaneous hyperglycemia	Type 2	- BM thickening in retinal capillaries	[65-67]
			- Loss of ECs and pericytes	
			- Acellular capillaries	
Otsuka Long-Evans Tokushima fatty rats	Spontaneous hyperglycemia	Type 2	- BM thickening in retinal capillaries	[68,69,71]
			- Microaneurysm formation	
			- Thinning of INL and PL	
Goto-Kakizaki rats	Spontaneous hyperglycemia	Type 2	- Apoptosis of ECs	[72-74]
			- Decreased retinal circulation	
Kimba mice	Transgenic overexpression of VEGF_165_ gene	N/A	- Increased vascular permeability	[77,78]
			- Retinal neovascularization	
OIR	Exposure to hyperoxia during early postnatal periods	N/A	- Increased vascular permeability	[47-49,79]
			- Retinal neovascularization	
Akimba mice	Crossing Akita mice with Kimba mice	Type 1	- Thinning of PL	[80]
			- Loss of ganglion cells in GCL	
			- Capillary nonperfusion	
			- Retinal neovascularization	
Diabetic Torii rats	Spontaneous hyperglycemia	Type 2	- Large retinal folds mimicking TRD	[81-83]
			- Massive hemorrhage in A/C	
			- Acellular capillaries	
			- Loss of pericytes	
**Dogs**				
Galactose-fed dogs	Feeding a 30% galactose diet	Type 2	- Acellular capillaries	[85,86]
			- Microaneurysm formation	
			- Intraretinal hemorrhage	
			- NVD	
OIR	Exposure to hyperoxia during early postnatal periods	N/A	- Retinal neovascularization	[50,87]
			- Peripheral retinal ischemia	
			- Vitreous hemorrhage	
			- Tractional retinal folds	
**Monkeys**				
Spontaneously diabetic	Spontaneous hyperglycemia	Type 2	- IRMA	[88,89]
			- Microaneurysm formation	
			- Retinal hemorrhage	

*A*/*C* Anterior chamber, *BM* Basement membrane, *EC* Endothelial cell, *GCL* Ganglion cell layer, *INL* Inner nuclear layer, *IPL* Inner plexiform layer, *IRMA* Intraretinal microvascular abnormality, *N*/*A* Not applicable, *NVD* New vessels at the optic disc, *OPL* Outer plexiform layer, *PL* Photoreceptor cell layer, *STZ* Streptozocin, *TRD* Tractional retinal detachment.

#### *Zebrafish*

Zebrafish are valuable tools for high throughput screening of candidate drugs and investigation of cell to cell interactions in normal or pathologic conditions. Particularly, it is relatively easy to handle multiple zebrafish at the laboratory facility because they are so tiny that occupy relatively small space. Furthermore, the introduction of fli1:EGFP zebrafish which show the fluorescent expression of vascular structures makes zebrafish-based models more attractive for the investigation of vascular abnormalities regarding pathologic conditions [36].

### High glucose-induced models

Zebrafish are known to demonstrate a similar metabolic regulation of glucose as human and mice [37]. Immersing the fish in 2% glucose solution every other day for 28 days induces hyperglycemia [38]. Generally, hyperglycemia possibly leads to apoptosis of neuronal and perivascular cells and atrophy of retinal layers. Interestingly, alternate exposure to glucose/water solutions results in thinning of the inner plexiform and inner nuclear layers (IPL and INL, respectively) and the ratio of IPL to INL is decreased by nearly 45% [38]. Furthermore, the same treatment demonstrates deleterious effects on cone photoreceptor cells, evidenced by definite degeneration of the photoreceptor cell layer (PL) and alterations in cone-mediated electroretinogram [39]. In this regard, this model helps researchers to dig out the cellular response to hyperglycemia and further pathologic changes in the diabetic retina. In addition, electron microscopy studies show that the width of interendothelial junctions and the thickness of BM in retinal capillaries are increased, suggesting the disruption of neurovascular units and inner BRB in this model [39]. As injection of tracers of different molecular sizes can be utilized for the demonstration of relative vascular permeability, we expect that this zebrafish model will be an animal model for high throughput screening of candidate drugs to control vascular permeability [36].

Streptozocin promotes cytotoxicity of β cells of pancreas to induce hyperglycemia in various animals including mice and rats [40-45]. Likewise, intraperitoneal injection of streptozocin in zebrafish leads to hyperglycemia and further characteristic findings corresponding to DR such as thinning of IPL and PL [46]. Such universal responses to hyperglycemia in zebrafish as mammals make DR models in zebrafish to be attractive animal models to investigate the pathogenesis of DR.

### Hypoxia-induced pathologic angiogenesis and vascular changes

In mice, rats, and dogs, oxygen-induced retinopathy (OIR) models are widely utilized for the investigation of retinal neovascularization [47-50]. Although these models do not show hyperglycemia, it is worth mentioning these models in this review on animal models of DR in that they are validated animal models of retinal neovascularization, the prominent clinical finding of PDR. Exposure to hyperoxia leads to the induction of cellular and histologic responses to hypoxia on returning to the normal condition. In this regard, OIR models can be utilized in studies on pathologic angiogenesis regarding hypoxia. Currently, there is no animal model using this concept in zebrafish. However, placing zebrafish in hypoxic water for 3 to 10 days results in retinal neovascularization, demonstrated by increased vascular sprouts in flat-mounted zebrafish retina [51,52]. As previously mentioned, vascular structures are easily visualized in fli1:EGFP zebrafish. Furthermore, with this model, researchers can quantitatively analyze the efficacy of candidate drugs which are diluted in the solution and systemically applied to zebrafish [51].

#### *Mice and rats*

Most of animal models of DR are based on rodents, mice and rats. We speculate that we cannot cover all published animal models and may skip some in this review because we intentionally include widely utilized and validated animal models. Interestingly, rodent models of DR are largely divided into 3 subgroups: 1) models with induced hyperglycemia, 2) spontaneously diabetic models, and 3) models with definite retinal neovascularization. The first 2 subgroups basically reflect structural abnormalities in DR without proliferative characteristics. In contrast, the latter is a valuable tool to investigate the molecular components of retinal neovascularization and therapeutic potential of candidate therapeutics in the treatment of PDR.

### Models with induced hyperglycemia

Streptozocin-induced death of pancreatic β cells leads to hyperglycemia in mice and rats. In mice, 1 or 2 days after the intraperitoneal injection of streptozocin, the level of serum glucose reaches above 350 mg/dl. Interestingly, the thickness of BM of retinal capillaries is increased in streptozocin-induced diabetic mice and rats [41,44]. Cell death of components of neurovascular units is also observed in both models. Neuronal cells in the ganglion cell layer (GCL) undergo apoptotic cell death in the streptozocin induced mice model [42]. In addition to neuronal cell death, chronic elevation of serum glucose induces degeneration and selective loss of pericytes of retinal capillaries in streptozocin-induced diabetic rats [43]. Furthermore, the number of amacrine cells is decreased in diabetic rats induced by streptozocin [45]. This loss of neuronal cells might be the reason of the phenomenon that certain layers of the diabetic retina are thinner than those in normal control.

**Figure 2:**
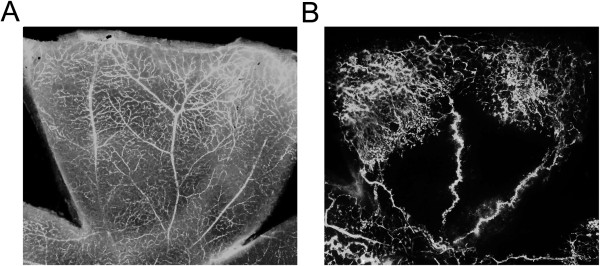
**Animal models of DR demonstrating increased vascular permeability** (**A**) **and retinal neovascularization** (**B**)**.** (**A**) Intraperitoneal injection of streptozocin to mice leads to hyperglycemia in 2 ~ 3 days and increased vascular permeability ensues in a week. Flat-mounted retina after the intravenous injection of FITC-dextran evidences diffuse leakage of retinal vessels. (**B**) Exposure to hyperoxia from P7 to P12 results in retinal neovascularization, which starts at P14 and peaks at P17. Flat-mounted retina after the intravenous injection of FITC-dextran demonstrates central ischemic retina with small vascular tufts at the junction between vascularized and avascular retina.

**Figure 3:**
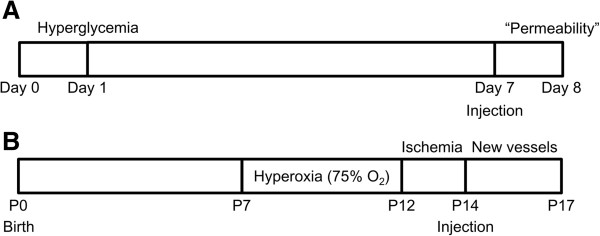
**Schematic protocols of the streptozocin**-**induced diabetic mice model** (**A**) **and the OIR mice model** (**B**)**.** (**A**) One or 2 days after the intraperitoneal injection of streptozocin to mice, the level of serum glucose reaches above 350 mg/dl. One week later, vascular permeability can be identified with the leakage of tracer molecules such as FITC-dextran. At day 7 from streptozocin injection, candidate drugs are injected intravitreally and the therapeutic effects of them can be analyzed 24 hours after the injection (day 8). (**B**) Newborn mice are exposed to hyperoxia from P7 to P12 and then returned to room air. Intravitreal injection of candidate drugs are performed at P14 and the therapeutic effects of them can be analyzed at P17.

As previously mentioned, increased vascular permeability and further ME are targets of therapeutic application in DR. Interestingly, streptozocin-induced hyperglycemia promotes vascular permeability in a week (Figure 2). After the induction of vascular permeability, suppressive effect of candidate drugs on vascular permeability is analyzed with the intravitreal administration of candidate drugs for 24 hours [29,35,53]. The schematic protocol of the streptozocin-induced hyperpermeability model is demonstrated in Figure 3.

### Spontaneously diabetic models

Certain strains of mice and rats demonstrate hyperglycemia during the lifetime. Full coverage of detailed characteristics of each model is beyond the purpose and scope of this review; therefore, we focus the introduction of related articles and the concept of proper selection of animal models according to the purpose of studies.

#### *db/db mice*

In this model, above-mentioned structural abnormalities such as loss of pericytes and BM thickening are observed [54-56]. Hyperglycemia starts at 8 weeks after the birth and mice over 18 weeks are usually utilized for the study investigating DR, because microvascular complications are demonstrated at this moment [57]. Interestingly, VEGF is increased in the vitreous fluid of this mouse model at 18 to 20 weeks [58]. Elevation of VEGF and other pathologic mechanisms such as oxidative stress might be inducing factors of vascular permeability in this model [55,59].

#### *Non-obese diabetic (NOD) mice*

In NOD mice, the cumulative incidence of diabetic phenomena such as polyuria, polydipsia, hyperglycemia, glycosuria, and hypercholesterolemia is 80% in females up to 30 weeks of age [60]. As other animal models of DR, this model demonstrates apoptosis of ganglion cells in GCL [61]. Hyperglycemia definitely affects the viability of neuronal and perivascular cells consisting of neurovascular units. Furthermore, focal vascular proliferation appears to protrude from the retinal surface into the vitreous cavity and vascular perfusion is decreased in retinas from NOD mice [62]. This characteristic might be due to increased expression of angiogenic factors such as VEGF and endothelin-1 [61,62].

#### *Akita (Ins2^Akita^) mice*

This model is characterized by progressive loss of pancreatic β cells and resultant hyperglycemia. Hyperglycemia starts to appear as early as 4 weeks, and microvascular abnormalities and ongoing retinal new vessels can be observed with maintenance of animals for longer duration [63,64]. Early diabetic changes in this model include acellular capillaries and loss of neuronal cells in GCL, pericytes, and amacrine cells [45,63,64]. Furthermore, increased vascular permeability is demonstrated by the increase in vascular leakage. Interestingly, after 9 months of observation, a characteristic finding of new vessel formation is observed in the outer plexiform layer [64]. Although retinal neovascularization usually exists in inner retinal layers in DR patients, the formation of retinal new vessels in this model is itself an interesting finding arousing researchers’ interests.

#### *Zucker diabetic fatty rats*

Genetic propensity for diabetes is only expressed in obese males in this model [65]. Usually, rats at approximately 20 weeks of age are utilized for the experiments, and control lean Zucker rats are examined for normal controls [65,66]. Alike with above-mentioned animal models, the thickness of BM of retinal capillaries is increased in this model [65,66]. Furthermore, loss of endothelial cells and pericytes around retinal capillaries is observed and these characteristics can be modified with the intravitreal injection of candidate drugs [67].

#### *Otsuka long-Evans Tokushima fatty rats*

From 5 months of age, this model demonstrates hyperglycemia and further microvascular complications of diabetes [68]. Due to loss of neuronal cells by hyperglycemia, INL and PL are thinned in this model. BM thickening in retinal capillaries is another characteristic feature of this model corresponding to pathologic changes in DR [68,69]. However, there is concern that this model is not suitable for the investigation of angiopathy in DR because the number of acellular capillaries and pericyte ghosts is not significantly increased [70]. Researchers who have much interest in these phenomenon should pay attention to this report, but considering the report that mRNA level of VEGF is increased in the retina of this model at 60 weeks of age, this model might be utilized for the investigation of roles of growth factors in the development of DR [71].

#### *Goto-Kakizaki rats*

This model is one of animal models mimicking type 2 diabetes. In DR, due to high osmolarity of blood, retinal circulation is expected to be decreased. Interestingly, retinal circulation time is prolonged in this model compared to that in normal controls [72]. Furthermore, VEGF expression of whole eyes is significantly increased, suggesting a role of VEGF in the development of pathologic changes in this model [73]. In addition, increased level of apoptosis of retinal microvascular cells suggests the cytotoxic effect of hyperglycemia on endothelial cells [74]. However, researchers should differentiate the consequences of hyperglycemia from inheritable pathologic changes not related to diabetes. Electroretinographic analysis of this model shows that hyperglycemia and further hypoxia do not affect functional abnormalities of photoreceptor cells, which are evident from 4 weeks of age [75]. Likewise, although WBN/Kob rats demonstrate hyperglycemia from 9 months of age, some researchers disregard them as animal models of DR because retinal changes of this model might be due to hereditary retinal degeneration [76].

### Models with definite retinal neovascularization

Unfortunately, most of above-mentioned animal models do not demonstrate retinal neovascularization, a characteristic finding in PDR patients. In this regard, researchers often utilize animal models with definite retinal neovascularization even though some of these models are not characterized by hyperglycemia. Therefore, we should pay more attention in the interpretation of data in these models to find relevant findings regarding DR.

#### *Kimba mice*

Through microinjection of a DNA construct containing the human VEGF_165_ gene, transgenic mice were generated [77]. Interestingly, pathologic features in this model include extensive retinal neovascularization, increased vascular leakage, and capillary non-perfusion, suggesting the roles of VEGF in the development of these vascular abnormalities [77,78].

#### *OIR mice and rats*

To induce retinal neovascularization in the OIR models, newborn rodents are exposed to hyperoxia during early developmental periods. The exact protocols are different from species to species, but the principal mechanism of animals is the same [47,49]. As for mice, newborn mice are placed in a chamber with 75% oxygen from postnatal day (P) 7 to P12. At P12, the central area of the retina experiences regression of retinal vessels and further ischemia [48]. Retinal neovascularization ensues at P14 and peaks at P17 [79]. With the intravenous injection of fluorescein isothiocyanate-dextran or immunofluorescent staining with endothelial cell markers such as isolectin-B4 or CD31, retinal neovascularization can be visualized qualitatively and analyzed quantitatively with the image processing program [48,79]. A representative figure of the retina of OIR mice and the schematic protocol of the induction of OIR in mice are demonstrated in Figure 2 and 3, respectively.

#### *Akimba mice*

Models with definite retinal neovascularization usually do not demonstrate hyperglycemia. Interestingly, crossing the Akita mice with Kimba mice, the Akimba mice (Ins2^Akita^VEGF^+/−^) were generated to demonstrate hyperglycemia and retinal neovascularization at the same time [80]. At 25 ± 1 weeks of age, Akimba mice show structural abnormalities such as thinning of PL and loss of ganglion cells. In addition, vascular pathology is more enhanced than parental strains, demonstrating severe capillary nonperfusion and retinal neovascularization. This model might be appropriate to be utilized in the investigation of the interaction between hyperglycemia and growth factors such as VEGF in the pathogenesis of DR.

#### *Diabetic Torii rats*

This model is interesting in that it shows pathologic features mimicking complications of PDR such as TRD and VH. Glycosuria starts at approximately 20 weeks of age and almost all male rats are evidenced to have diabetes at 40 weeks of age [81]. Interestingly, histopathological studies show TRD with fibrous proliferation and massive hemorrhage in the anterior chamber in this model [81,82]. Furthermore, this model also demonstrates structural abnormalities such as acellular capillaries and pericyte loss [82]. There is a significant reduction in amplitudes and prolongation in implicit times of a-wave, b-wave, and oscillatory potential with the aging and progression of diabetes in the electroretinographic analyses [83]. However, there are definite differences between retinal neovascularization in diabetic Torii rats and that in human PDR in that this model develops retinal new vessels without prominent retinal ischemia [84]. Possibly, increase in angiogenic factors not from activation by ischemic insults might be the cause of retinal neovascularization in this model.

#### *Dogs*

Compared to zebrafish, mice, and rats, dogs are more difficult to manipulate and maintain for the experiments. Furthermore, as they are basically companion animals in many cultural areas, there is much emotional resistance to utilize dogs as laboratory animals. However, in the development process of candidate therapeutics and the investigation of pathologic events in DR, canine animal models have several advantages as follows: 1) Dogs have the macula which zebrafish, mice, and rats do not have. In this regard, dogs have more similarity in the structure of the retina with human. 2) The size of eyeball is almost the same with human. Therefore, studies on pharmacokinetic characteristics and various administration methods are possible with canine animal models. 3) Furthermore, machines for the evaluation of the retinal structure and function such as optical coherence tomography and electroretinography which are clinically utilized can be easily applied to canine animal models.

#### *Galactose-fed dogs*

Galactose feeding results in pathologic changes corresponding to human DR in beagles [85,86]. Interestingly, fluorescein angiography shows many characteristic findings of human DR in the canine galactose-feeding model such as IRMA, microaneurysm, intraretinal hemorrhage and NVD. A weak point of this model for widespread utilization might be long feeding periods for the induction of diabetes (more than 28 months) [85]. However, this model can be attractive to researchers who want to utilize canine animal models for the DR research.

#### *OIR dogs*

Similarly to OIR models in mice and rats, newborn beagles are exposed to 95% to 100% oxygen continuously for 4 days and returned to room air [50,87]. In this model, retinal neovascularization with incomplete vascularization of peripheral retina occurs to develop VH and tractional retinal folds [50].

#### *Monkeys*

Type 2 diabetes occurs spontaneously in obese rhesus monkeys [88,89]. As the ocular structure of rhesus monkeys are nearly same with that of human, microanigopathic pathology in these monkeys looks similar to that in DR patients. Pathologic findings in this model include cotton-wool spots, intraretinal hemorrhages, hard exudates, dot/blot hemorrhage, IRMA, and microaneurysms. All of these features are exactly those in patients with NPDR.

## Conclusions

### Purpose-based selection of appropriate animal models

Animal models have deepened our knowledge about the pathogenesis of diseases and led the development of valuable therapeutics to overcome pathologic consequences of diseases. As for DR, we also owe advancements in the investigation to validated animal models. However, even validated animal models of DR have limitations. Some do not demonstrate characteristics corresponding to PDR, and others require too much time to develop pathologic features of DR, making the process of the evaluation of the therapeutic efficacy of candidate drugs tedious. To investigate complex interactions among different types of cells and multiple pathologic mechanisms, it is not appropriate to utilized animal models based on modification of a single factor. For example, intravitreal injection of VEGF only might be not an appropriate model for such a study. Likewise, researchers should utilize animal models with retinal neovascularization, not merely with induced hyperglycemia without new vessels, to evaluate the therapeutic potential of candidate drugs on PDR. Furthermore, researchers can select animal models with vascular permeability such as mice with induced hyperglycemia (by streptozocin injection), db/db mice, Akita mice in the process of drug development for ME.

Animal models of DR are based on hyperglycemia, hypoxia, or introduction of VEGF gene. In this regard, these animal models can be utilized to investigate pathogenesis and potential therapeutics regarding these pathologic conditions. We expect that proper selection of animal models according to the purpose of studies might improve the potency of DR research to help to minimize the risk of visual deterioration in DR patients from vision-threatening complications.

## Abbreviations

A/C: Anterior chamber; AMD: Age-related macular degeneration; BM: Basement membrane; BRB: Blood-retinal barrier; CSME: Clinically significant macular edema; DR: Diabetic retinopathy; EC: Endothelial cell; ETDRS: Early treatment of diabetic retinopathy Study; GCL: Ganglion cell layer; INL: Inner nuclear layer; IPL: Inner plexiform layer; IRMA: Intraretinal microvascular abnormality; IVTA: Intravitreal administration of triamcinolone acetate; ME: Macular edema; N/A: Not applicable; NOD: Non-obese diabetic; NPDR: Nonproliferative diabetic retinopathy; NVD: New vessels at the optic disc; OIR: Oxygen-induced retinopathy; PDR: Proliferative diabetic retinopathy; PL: Photoreceptor cell layer; PRP: Panretinal photocoagulation; STZ: Streptozocin; TRD: Tractional retinal detachment; VEGF: Vascular endothelial growth factor; VH: Vitreous hemorrhage.

## Competing interests

The authors declare that they have no competing interests.

## Authors’ contributions

DHJ designed the concept, collected information, and prepared the manuscript and figures. CSC and HOJ prepared the figures. JiHK contributed the discussion and edited the manuscript. JeHK designed the concept and edited the manuscript. All authors read and approved the final manuscript.
